# Social Inhibition and Depressive Symptoms among Couples with Children with Autism Spectrum Disorder: The Mediating Role of Perceived Family Support

**DOI:** 10.3390/medicina60030488

**Published:** 2024-03-15

**Authors:** Ting Pan, Tinakon Wongpakaran, Nahathai Wongpakaran, Bijing He, Danny Wedding

**Affiliations:** 1 Master of Science Program (Mental Health), Multidisciplinary and Interdisciplinary School (MIdS), Chiang Mai University, Chiang Mai 50200, Thailand; ting_pan@cmu.ac.th (T.P.); nahathai.wongpakaran@cmu.ac.th (N.W.); bijing_he@cmu.ac.th (B.H.); danny.wedding@gmail.com (D.W.); 2 Department of Psychiatry, Faculty of Medicine, Chiang Mai University, Chiang Mai 50200, Thailand; 3 Department of Clinical and Humanistic Psychology, Saybrook University, Pasadena, CA 91103, USA; 4 Department of Psychology, University of Missouri-Saint Louis, St. Louis, MO 63121, USA

**Keywords:** social inhibition, autism, depression, family support, parents, actor–partner interdependence mediation model

## Abstract

*Background and Objectives*: A limited understanding exists regarding the intricate dynamics between the levels of social inhibition exhibited by both wives and husbands concerning their perceived family support and depressive symptoms, particularly within couples who are parents of children diagnosed with autism spectrum disorder (ASD). *Materials and Methods*: This study used the actor–partner interdependence mediation model to analyze data collected from 397 pairs of Chinese parents with children diagnosed with ASD. *Results*: The findings of the study revealed significant indirect actor effects, indicating that the levels of social inhibition exhibited by both wives and husbands were associated with their own depressive symptoms through their respective perceptions of family support. In general, the study did not find significant partner effects, except for some indirect effects of wives on their husbands’ depressive symptoms through the wives’ perceived social support. *Conclusions*: In line with related studies, social inhibition was associated with depressive symptoms. At the same time, perceived family support could be a mediator of depression. Gender differences in emotional expression, influenced by cultural norms and distinct role expectations within the family context, may elucidate why only wives’ perceived family support could impact husbands’ depressive symptoms. These results underscore the potential importance of interventions aimed at addressing social inhibition and enhancing perceived family support to alleviate depressive symptoms in this population. Additionally, encouraging family support for both wives and husbands’ involvement in collaboration may be of benefit in improved outcomes for both parents and children within families affected by ASD.

## 1. Introduction

According to DSM-5, autism spectrum disorder (ASD) constitutes a group of neurodevelopmental disorders with the core symptom of social dysfunction, as well as limited and repetitive stereotyped behaviors [1]. It directly affects its patients and indirectly affects their parents, experiencing the stigma attached to the disease. According to a Western study, parents of children with ASD are more likely to experience depression than those of children without ASD, with 34.2% of parents with children with ASD presenting clinical depressive symptoms [2]. Regarding the Chinese research, the distribution rate ranges from 25 to 31% [3,4].

Parents of children with ASD usually experience distress, anxiety, depression, economic pressure, a high divorce rate, and lower family well-being compared with those whose children have other developmental conditions [5,6,7,8]. Differences have been noted in the depression distribution rate between fathers and mothers of children with ASD, in that 50% of mothers with children with ASD are depressed compared with 17% of mothers without children with ASD, whereas only 21% of fathers of children with ASD are depressed compared with 15% of fathers without [9]. Mothers are most affected, with about one-third of mothers experiencing depression [10]. When parents’ depression continues unrelieved, it may further aggravate the condition of children with ASD [11]. 

Many factors are involved in developing depression among parents of children with ASD. One of these is social inhibition, a tendency characterized by personality sensitivity (e.g., fear of negative evaluation), social withdrawal (e.g., social interaction avoidance), and behavioral inhibition (e.g., difficulty in communicating with others). Individuals with social inhibition may have difficulty regulating their emotions, which negatively impacts their mental and physical health [12]. Research has shown that social inhibition is a risk factor for depression; on the other hand, depression can aggravate social avoidance problems as well [13,14,15,16,17].

Even though social inhibition is viewed as a trait, social inhibition behavior can occur among parents of autistic children [13,18,19]. They may avoid taking their children to public places or participating in some social activities to reduce the social pressure caused by their children’s abnormal behaviors. They may be sensitive to the social information conveyed by others and the perceived rejection and unfriendly treatment of others [20,21,22].

Social support, on the other hand, is significantly associated with decreased depressive symptoms. Mothers of children with ASD suffer blame and exclusion from other relatives, leading to a lack of social support and family member support and loneliness [23]. Researchers have revealed that family support can be a protective factor against depressive symptoms [24,25,26,27], and limited social support is associated with depression among parents of children with ASD [7]. Furthermore, perceived family support could be a mediator of depression [28]. The relationship between social inhibition and perceived social support has been demonstrated [15]. A study of patients with tumors showed that social inhibition influenced their perception of social support [29].

Regarding the relationships among social inhibition, family support, and depression, a study showed that perceived social support mediated the relationship between a distressed personality (e.g., negative feelings and social inhibition) and depression [30]. However, there is a lack of research on the intermediary role of family support in the relationship between social inhibition and depression among couples who are parents of children with ASD. Moreover, regarding relationships where partner effects influence the outcome, no research has been carried out concerning the mediating role of family support on social inhibition and depression among couples who are parents of children with ASD within a dyadic framework.

The actor–partner interdependence mediation model (APIMeM) makes it possible to estimate intermediary, indirect, and direct effects, especially when dyad members are distinguishable (such as in heterosexual couples), creating an understanding of the unique contributions of the parents’ own social inhibition to their own (actor effect) and each other’s (partner effect) depressive symptoms, while their own family support mediates this relationship [31].

This study aims to explore how wives and husbands’ own social inhibition affects their own (actor effect) and each other’s (partner effect) depressive symptoms while their own perceived family support mediates the relationship, which can benefit further research on the depression treatment for them. This study using the APIMeM conceptual framework hypothesized that individual (actor) perceived family support would mediate the relationship between social inhibition and depressive symptoms for both wife and husband. In addition, it was hypothesized that the partner effect would be observed. 

**Figure 1:**
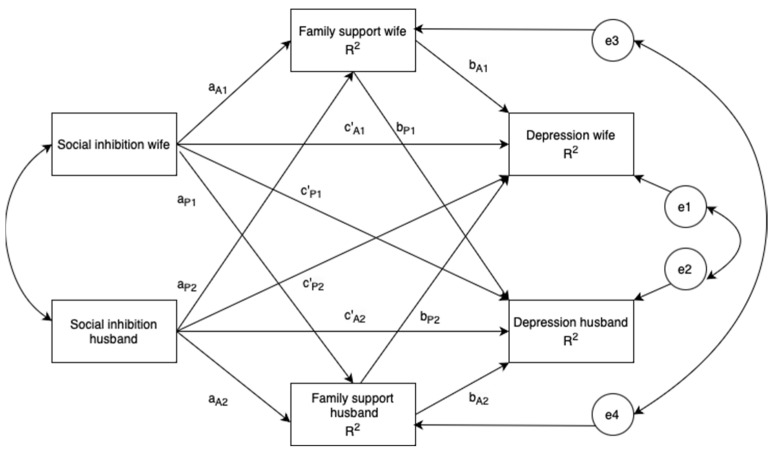
A hypothetical actor–partner interdependence mediation model in couples who are parents of ASD children. a, direct effect of social inhibition on perceived family support; b, direct effect of perceived family support on depression; c′, direct effect of social inhibition on depression; A1 and A2, actor effect; P1 and P2, partner effect; e_1_, e_2_, e_3_, and e_4_, latent error terms; *R*^2^, coefficient of determination; 1, wife; 2, husband. Estimates are unstandardized regression coefficients. Significant path coefficients are in red.

The hypotheses to be tested for each effect of the APIMeM, such as actor–actor effect, actor–partner effect, partner–actor effect, and partner–partner effect, are presented below (Figure 1). 

Actor–actor effect: A wife’s and husband’s social inhibition would be associated with their own depression through the mediating effect of their own family support. 

Actor–partner effect: A wife’s and husband’s social inhibition would be associated with their own perceived family support, which, in turn, would be negatively associated with the depression of their counterpart. 

Partner–actor effect: A wife’s and husband’s social inhibition would be associated with the perceived family support of their counterpart, which, in turn, would be negatively associated with the depression of their counterpart.

Partner–partner effect: A wife’s and husband’s social inhibition would be associated with the perceived family support of their counterpart, which, in turn, would be negatively associated with their own depression.

There are few research works studying the mediating role of family support between social inhibition and depression among parents with ASD children. In terms of theoretical significance, this article can help fill the research gap on depression in parents with ASD children. It can also be studied from a new perspective of whether and how family support mediates the relationship between social inhibition and depression in a dyadic framework. In terms of practical significance, it is beneficial to understand the psychological process of depression in parents of children with ASD. Social inhibition and perceived family support may be treatments to reduce their depression. 

## 2. Materials and Methods

The study utilized a cross-sectional survey design involving couples who were parents of children with ASD. The sample consisted of 397 pairs of parents, comprising 397 wives and 397 husbands, with ages ranging from 23 to 45 years old in mainland China. Ethical approval for this research was obtained from the Research Ethics Committee, the Faculty of Medicine, Chiang Mai University (Approval Number: PSY-2566-0324).

### 2.1. Participants

The participants comprised general Chinese parents with children diagnosed with ASD. An online survey was employed as the chosen means to generate the invitations and gather data. The inclusion criteria included (1) being spouses participating as a pair, (2) having one or more children with diagnosis of ASD, and (3) being able to read and write Chinese proficiently and independently complete the research questionnaire. The exclusion criteria included one spouse refusing to participate and individuals being unable to participate online. Upon completion of the socio-demographic information, the participants proceeded with the subsequent measurements described below.

### 2.2. Study Measures

#### 2.2.1. Demographic Data on Parents’ and Children’s Demographic Information

These included age, number of children, educational background, occupation, living area, income, expense, time in childcare, marital status, and duration of marriage.

#### 2.2.2. Inventory for Interpersonal Problems (IIP)—Social Inhibition Subscale

IIP is a self-reporting questionnaire which measures interpersonal difficulties across eight sub-scales, including those derived from the following dimensions: affiliation (from hostile/cold to friendly behavior) and domineering (from submissive to controlling behavior). In this study, only the social inhibition subscale was used (for example, “I am too afraid of other people”) [32]. A higher score indicates a higher level of social inhibition. Cronbach’s alpha coefficient was 0.84 in the Chinese version [33]. In this study, Cronbach’s alpha was 0.82. See the Supplementary Materials: Inventory S1.

#### 2.2.3. Multidimensional Scale for Perceived Social Support (MSPSS)—Family Support Subscale 

The MSPSS is a self-reporting questionnaire designed to assess the extent to which an individual perceives support from family members. It comprises 4 items, each rated on a 7-point Likert scale [34]. Higher scores indicate a greater perceived level of support. The Chinese version of the MSPSS has been validated and shown to be reliable [35]. In our study, Cronbach’s alpha coefficient for internal consistency was calculated to be 0.86. See Supplementary Inventory S2.

#### 2.2.4. Core Symptom Index (CSI)—Depression Subscale

CSI—Depression is a self-reporting questionnaire designed to assess the extent to which an individual perceives their own depression. It consists of a 5-item, 5-point Likert-type scale ranging from 0 (not at all) to 4 (always) [36]. A higher score indicates a higher level of depressive symptoms. Cronbach’s alpha for the depression subscale was 0.85. In our study, Cronbach’s alpha coefficient for internal consistency was calculated to be 0.87. See Supplementary Inventory S3.

### 2.3. Statistical Analysis

Descriptive analysis was applied to the demographic data and social inhibition, family support, and depression scores. Covariates were analyzed using numbers and percentage. Pearson’s correlation was used to test the correlation and the direction of the correlation among the variables. Descriptive and Pearson’s correlation analyses were conducted using IBM SPSS version 26.0. 

Before conducting the mediation analysis, a significant association between social inhibition (X) and depression (Y) should be established, as well as a significant association between perceived family support (M) and both X and Y. In addition, potential confounding or third variables that could influence both the mediator and the outcome were controlled to ensure that any observed mediation effects were not spurious. Mediation or indirect effects were analyzed using bootstrapping approaches to ensure accuracy. The Hayes approach identified significant indirect effects in establishing the mediation model [37].

Next, we applied the actor–partner interdependence mediation model (APIMeM) using Amos 26.0 to investigate the actor and partner effects on the relation between depression and social inhibition as they were mediated by perceived family support. This model aimed to investigate the direct actor and partner effects and indirect actor and partner effects, including the actor–actor effect, actor–partner effect, partner–actor effect, and partner–partner effect.

Within the actor–partner interdependence mediation model, social inhibition’s effect on perceived family support is denoted as a, perceived family support’s effect on depression as b, and social inhibition’s direct effect on depression as c’. Subsequently, the total effect (TE) (ab + c’), the mediating effects (ab), and the direct effects (a, b, and c’) were computed (Table 1). The mediating effect was estimated as the multiplication of the direct effects a and b, indicating the portion of the relation between social inhibition and depression mediated by perceived support from family members. Meanwhile, the TE was calculated (c’ + ab), representing the association between social inhibition and depression before adjusting for support from family members. The APIM analysis was conducted using structural equation modeling in AMOS 22 and IBM SPSS version 26 (IBM Corp, Armonk, NY, USA) with a dyadic dataset. To test the *p* value of the total and mediating effects, a 95% confidence interval corrected for bias using Monte Carlo sampling was used to bootstrap sample 5000. The overall fitness of the APIMeM model was evaluated using the model fit index, including the CFI, TLI, RMSEA, and SRMR. All statistical analyses are two-tailed, and a *p* value less than 0.05 was used.

## 3. Results

### 3.1. Sociodemographic Results

In total, 794 participants were included in the data analysis, consisting of 397 wives and 397 husbands. While the wives’ ages ranged from 26 to 43 years old, the husbands’ ages varied from 23 to 45 years old. Overall, the wives tended to be younger than the husbands (*p* < 0.001). The greatest proportion had earned university degrees (47.3%), were employed (94.1%), resided in urban areas (68.3%), and reported a household monthly income exceeding 5000 CNY (99.2%). Additionally, most of the families incurred an annual cost of approximately 30,000 CNY for the care of a child with ASD (81.6%) and dedicated at least 6 h daily to parenting duties (72.7%), with the wives typically spending more time on parenting (*p* < 0.001). Regarding marital duration, a significant portion of the participants reported being married for over ten years. Further details can be found in Table 2.

Table 3 displays the psychosocial variables. There were no significant differences in the mean scores between wives and husbands, except for depressive symptoms, where the wives scored significantly higher than the husbands (*p* = 0.001).

### 3.2. Correlation Results

The findings revealed a significant positive correlation between both the wives’ and husbands’ levels of social inhibition and their respective depressive symptoms (*r* = 0.338 to 0.405, *p* < 0.01). In contrast, there existed a negative correlation between perception of family support and social inhibition (*r* = −0.154, *p* < 0.01), as well as between their own and their partner’s depressive symptoms (*r* = −0.077 to −0.311, *p* < 0.05). Notably, there was a positive association between husbands’ social inhibition and their partners’ depressive symptoms (*r* = 0.71, *p* < 0.05), along with wives’ depression being positively correlated with their husbands’ depression (*r* = 0.257, *p* < 0.01) (Table 4).

### 3.3. APIMeM Analysis Results (Standardized Estimates)

Overall, our analysis revealed actor effects for both wives and husbands, indicating direct or indirect impacts on their own depressive symptoms, while partner effects were not evident. Specifically, both a wife’s and a husband’s depression were predicted by their own levels of social inhibition (*β* = 0.290–0.362, *p* ≤ 0.001) and perceived family support (*β* = −0.225 to −0.212, *p* < 0.001). Additionally, their own social inhibition was associated with their perceived family support (*β* = −0.154–0.153, *p* < 0.001). Interestingly, husbands’ depression was significantly influenced by their wives’ perceived family support (*β* = −0.071, *p* = 0.031). However, husbands’ social inhibition (*β* = 0.041, *p* = 0.235) and perceived family support (*β* = 0.042, *p* = 0.197) were not significantly associated with wives’ depression.

**Figure 2:**
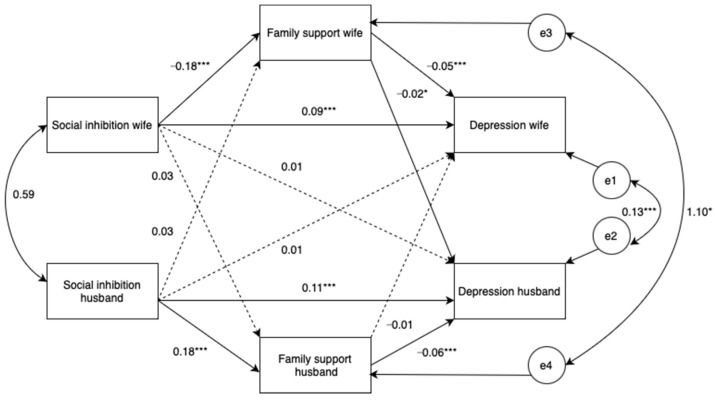
The actor–partner independence mediation model (unstandardized). All covariates, i.e., age, family income, and time spent on childcare, were included in the model (not shown). The R-squared values for wives’ and husbands’ depression were 0.311 and 0.263, respectively. Model fit statistics were as follows: Chi-square = 0.985; df = 1; *p* = 0.321; comparative fit index (CFI) = 1.000; Tucker–Lewis index (TLI) = 1.001; root mean square error of approximation (RMSEA) = 0.000; 90% confidence interval (90% CI) = (0.000–0.094); standardized root mean squared residual (SRMR) = 0.0037, * *p* < 0.05, *** *p* < 0.001.

Moreover, significant correlations were observed between wives’ and husbands’ perceptions of family support (*β* = 0.09, *p* = 0.15), as well as between the wives’ and husbands’ levels of social inhibition (*β* = 0.21, *p* < 0.001). The R-squared values for the entire model were 0.060 for the wives’ perceived family support and 0.061 for the husbands’ perceived family support. Regarding the IEs, both wives’ and husbands’ social inhibition were associated with their own depression through their perceived family support (*β* = 0.010, *p* < 0.001). Additionally, the wives’ social inhibition could influence their husbands’ depression through wives’ perceived family support (*β* = 0.003, *p* = 0.018). However, the social inhibition of either the wives or husbands did not exhibit a significant TE on their partner’s depression (*β* = 0.012, *p* = 0.248–0.259). Moreover, wives’ and husbands’ depression was not influenced by their own or their partner’s social inhibition through their partner’s perceived family support. Essentially, no partner–partner or partner–actor effects were observed for either wives or husbands (*β* = −0.002 to 0.000, *p* > 0.05) (Table 5 and Figure 2).

## 4. Discussion

The aim of the present study was to investigate how the social inhibition of couples might impact either their own or their partner’s experience of depressive symptoms, with “perceived family support” serving as a mediating factor among parents with children receiving a diagnosis of ASD within a dyadic framework. Our research revealed that the link between social inhibition and depression in the parents of children with ASD was influenced by their perceived family support. However, we only observed actor effects and not partner effects in the associations among social inhibition, perceived social support, and depressive symptoms among couples.

The findings of the current study improve our understanding of these parents’ mental health, how social inhibition contributes to perceived family support, and how perceived family support is associated with the depression of wives and husbands when parenting their children with ASD in Chinese couples. The results revealed that parents’ social inhibition was both directly and indirectly correlated with their own depression. This suggests that Chinese families with children with ASD may benefit from inventions targeting reduced social inhibition and family support.

Regarding the actor effect in the dyadic analysis using the APIMeM, it confirms our hypothesis that high levels of social inhibition would be associated with lower levels of perceived family support, and lower perceived family support was positively associated with depression for both wives and husbands who were parents of children with ASD. These findings are supported by some related studies that social inhibition is associated with depression among mothers of children with ASD [18], and perceived family support was a common mediator of depression [28]. Raising children with ASD may limit parents’ interpersonal relationships and social lives due to stigma, which may make these parents more withdrawn and anxious about interpersonal communication and efficiently contribute to their social inhibition [38]. Parenting children with ASD itself takes these parents a great deal of time, so they will be less involved in educational research, community activities, and public affairs [10]. As we know, social inhibition may affect individuals’ poor perception of family support [24]. These parents may perceive lower family support due to their exacerbated social inhibition, leading to depression to some extent.

However, the partner effect was not as significant as the actor effect, which was against our hypothesis based on family system theory, which posits that a family member’s emotions and behaviors may affect his/her partner [39]. This nonsignificant dyadic level was supported by a related study on emotional stress and dysregulation among parents of individuals with ASD in China [40].

Interestingly, even though there was a significant indirect effect of wives on their husbands’ depressive symptoms through the wives’ perceived family support, the total effect of this partner effect was still not significant. In other words, a partner’s perceived family support does not appear to affect an individual’s depression.

The fact that one partner’s social inhibition and perceived family support may not contribute to the other partner’s depression could be related to the fact wives and husbands have distinct roles in daily family life in traditional Chinese culture and most cultures around the world. By that, we mean that wives are expected to be the main caregivers of family members and of disabled people in the family [27,41,42]. While wives have this burden in caring for family members, husbands are expected to provide financial support and some assistance in childcare [43]. It is important to note that in the present study, the wives exhibited higher levels of depression and lower levels of perceived family support. Although one spouse may have felt supported by family, the potential impact on their partner was not shown. The reason for this may be because it was unclear from whom such support originated. Support from other family members, such as grandparents, may not have a significant effect on an individual’s depression compared to spousal support [44,45]. It is plausible that, in this case, the level of spousal support among families with children with ASD was low.

Although a small indirect effect was observed in wives’ social inhibition having a potential impact on husbands’ depression through wives’ perceived family support, this effect was not statistically significant. It is proposed that wives’ perception of inadequate family support could lead to their withdrawal, thereby contributing to their husbands’ unhappiness. Conversely, no such pathway was observed from husbands to wives, indicating distinct gender roles within Chinese families. Nonetheless, the absence of a partner effect may be associated with individuals’ social inhibition, particularly considering that Chinese mothers and fathers typically exhibit introverted and implicit emotional interactions [46]. In addition, a study has shown that parents with ASD children have hypersensitivity to criticism, anxiety, and aloofness [47], and these traits may be exacerbated in stressful families with ASD.

### 4.1. Research Implication

To our knowledge, this study represents the initial investigation into the impact of social inhibition on depressive symptoms among ASD parents from the perspective of perceived family support. Nonetheless, we urge further exploration into intervention studies, such as the provision of family or couple support, which can be more precisely identified.

### 4.2. Clinical Implication

Raising a child with autism spectrum disorder (ASD) can be a daunting task for parents. To prevent depression, it is crucial for couples to receive support from their partners and family members. Creating effective support networks through strategic programs is recommended. Furthermore, addressing social inhibition requires a multifaceted approach, which includes screening couples for factors that may influence this trait. By identifying and addressing these factors and improving communication, couples can navigate the challenges of raising a child with ASD and strengthen their relationship.

### 4.3. Limitations

Firstly, the reliance on self-reported measures introduces the possibility of biases, such as social desirability and response biases, which may impact the accuracy of the findings. Secondly, important covariates such as parental stress were not included in the analysis, potentially confounding the relationship between the variables under investigation. Thirdly, the cross-sectional design of the study limits our ability to establish causal relationships or determine the directionality of the associations among the examined variables. Future research employing longitudinal designs is recommended to elucidate the temporal dynamics of these parenting constructs and their impact on depressive symptoms over time.

Additionally, the generalizability of the findings may be limited by the study’s focus on married parents residing in mainland China. Other family structures and cultural contexts may yield different patterns of association between family support, social inhibition, and depressive symptoms. Therefore, caution should be exercised when extending these findings to diverse cultural and social contexts, including Chinese populations outside of mainland China and individuals from Western cultures.

Lastly, the exclusion of spousal support as a variable of interest in this study represents a notable limitation. Given its potential relevance to partners’ depression, future research should explore the role of spousal support in mitigating depressive symptoms within the context of parenting children with ASD.

## 5. Conclusions

This study’s findings suggest the presence of actor effects but not partner effects in the relationships among couples’ social inhibition, perceived social support, and depressive symptoms. This study makes several contributions to the existing literature. Firstly, it is one of the first studies to explore the mechanisms underlying the associations between social inhibition and depressive symptoms, incorporating both partners’ perceptions of social support as mediators. Secondly, by employing dyadic analysis, it allows for an investigation into the potential family processes influencing these relationships. Thirdly, our results highlight the importance of providing couples with skills to effectively offer and receive family support in mitigating social inhibition. Additionally, interventions targeting fathers should emphasize their active involvement in parenting and collaboration with their partners in caring for children with ASD, potentially leading to improved outcomes for both parents and children in ASD-affected families. The study also acknowledges its own limitations and offers suggestions for future research.

## Figures and Tables

**Table 1:** Calculation for the total effect (TE), direct effect, and indirect effects (IEs) in the actor–partner interdependence mediation model.

Actor Effect (Individual’s social inhibition → individual’s depression)			
Wife	Total effect	aA1 × bA1 + aP1 × bP2 + c’A1	Wife actor TE
	Indirect effect	aA1 × bA1 + aP1 × bP2	Wife actor total IE
	Actor–actor simple IE	aA1 × bA1	Wife actor–actor IE
	Partner–partner simple IE	aP1 × bP2	Wife partner–partner IE
	Direct effect	c’A1	Wife actor direct effect
Husband	Total effect	aA2 × bA2 + aP2 × bP1 + c’A2	Husband actor TE
	Total IE	aA2 × bA2 + aP2 × bP1	Husband actor total IE
	Actor–actor simple IE	aA2 × bA2	Husband actor–actor IE
	Partner–partner simple IE	aP2 × bP1	Husband partner–partner IE
	Direct effect	c’A2	Husband actor direct effect
Partner Effect (Individual’s social inhibition → partner’s depression)			
Wife	Total effect	aA1 × bP1 +aP1 × bA2 + c’P2	Wife partner TE
	Total IE	aA1 × bP1 +aP1 × bA2	Wife partner total IE
	Actor–actor simple IE	aA1 × bP1	Wife actor–partner IE
	Partner–actor simple IE	aP1 × bA2	Wife partner–actor IE
	Direct effect	c’P2	Wife partner direct effect
Husband	Total effect	aA2 × bP2 + aP2 × bA1 + c’P1	Husband partner TE
	Indirect effect	aA2 × bP2 + aP2 × bA1	Husband partner total IE
	Actor–partner simple IE	aA2 × bP2	Husband actor–partner IE
	Partner–actor simple IE	aP2 × bA1	Husband partner–actor IE
	Direct effect	c’P1	Husband partner direct effect

**Table 2:** Sociodemographic characteristics of the participants.

Variable	Family (*n* = 794)	Wives (*n* = 397)	Husbands (*n* = 397)	Test Difference
Age (years), mean (*SD*)		35.35 (3.07)	36.33 (3.36)	*t* = 4.29, *p* < 0.001
Number of children *n* (%)				
1	284 (71.5)			
2	112 (28.2)			
3	1 (0.3)			
Educational background *n* (%)				*χ*^2^ (4) = 15.89, *p* = 0.003
Elementary		3 (0.8)	4 (1)	
Junior high school		62 (15.7)	28 (7.1)	
High school		157 (39.6)	157 (39.6)	
Bachelor’s		171 (43.2)	204 (51.5)	
Master’s		3 (0.8)	3(0.8)	
Occupation *n* (%)				*χ*^2^ (1) = 45.80, *p* < 0.001
Unemployed or housekeeper		46 (11.6)	1 (0.3)	
Employed		351 (88.4)	396 (99.7)	
Living area *n* (%)				
Urban	271 (68.3)			
Rural	126 (31.7)			
Monthly income a (CNY) *n* (%)				*χ*^2^ (3) = 119.57, *p* < 0.001
0–3000		71 (18)	7 (1.8)	
3001–6000		205 (52)	142 (35.8)	
6001–10,000		112 (28.4)	202 (50.9)	
>10,000		6 (1.5)	45 (11.3)	
Family’s monthly earnings (CNY) *n* (%)				*χ*^2^ (3) = 0.25, *p* = 0.969
0–5000	3 (0.8)			
5001–10,000	158 (39.8)			
10,001–15,000	105 (26.4)			
>15,000	131 (33.0)			
Childcare expenses for child(ren) with ASD (CNY/year) *n* (%)				*χ*^2^ (3) = 0.04, *p* = 0.998
None	15 (3.8)			
1–30,000	324 (81.6)			
30,001–60,000	49 (12.3)			
>60,000	9 (2.3)			
Time spent caretaking for children each day (hours) *n* (%)				*χ*^2^ (3) = 176.66, *p* < 0.001
0–3		40 (10.1)	176 (44.3)	
6–8		165 (41.7)	174 (43.8)	
9–10		119 (30.1)	38 (9.6)	
>10		72 (18.2)	9 (2.3)	
Duration of marriage (years) *n* (%)				*χ*^2^ (3) = 0.27, *p* = 0.965
3–5		4 (1)	3 (0.8)	
6–8		5 (1.3)	4 (1)	
9–10		39 (9.8)	39 (9.8)	
>10		349 (87.9)	351 (88.4)	

**Table 3:** Mean and standard deviation of the psychosocial variable.

Variables	Wives (M ± SD)	Husbands (M ± SD)	Test Difference
Social inhibition	6.26 (3.06)	6.21 (3.20)	*t* = −0.23, *p* = 0.821
Perceived family support	18.03 (3.77)	18.14 (3.60)	*t* = 0.45, *p* = 0.656
Depressive symptoms	1.40 (0.89)	1.20 (0.88)	*t* = −3.21, *p* = 0.001

**Table 4:** Correlation analysis among psychological variables.

Variable	1	2	3	4	5	6
1. Wives’ social inhibition	-					
2. Husbands’ social inhibition	0.06	-				
3. Wives’ perceived family support	−0.154 **	0.01	-			
4. Husbands’ perceived family support	0.01	−0.154 **	0.092 **	-		
5. Wives’ depression	0.338 **	0.071 *	−0.310 **	−0.077 *	-	
6. Husbands’ depression	0.069	0.405 **	−0.109 **	−0.311 **	0.257 **	-

** *p* < 0.01, * *p* < 0.05.

**Table 5:** The total effect (TE), direct effect, and indirect effects (IEs) in the actor–partner interdependence mediation model (unstandardized).

Actor Effect (Individual’s social inhibition → individual’s depression)				
		coefficient	95% CI	*p* value
Wife	Total effect	0.095	(0.071, 0.117)	0.001
	Indirect effect	0.009	(0.004, 0.017)	<0.001
	Actor–actor simple IE	0.010	(0.004, 0.017)	<0.001
	Partner–partner simple IE	0.000	(−0.002, 0.000)	0.317
	Direct effect	0.086	(0.062, 0.108)	0.001
Husband	Total effect	0.118	(0.095, 0.141)	<0.001
	Total IE	0.010	(0.004, 0.018)	0.001
	Actor–actor simple IE	0.010	(0.005, 0.018)	<0.001
	Partner–partner simple IE	0.000	(−0.003, 0.001)	0.371
	Direct effect	0.108	(0.084, 0.131)	<0.001
Partner Effect (Individual’s social inhibition → partner’s depression)				
Wife	Total effect	0.012	(−0.009, 0.031)	0.259
	Total IE	0.002	(−0.005, 0.008)	0.577
	Actor–actor simple IE	0.003	(0.001, 0.008)	0.018
	Partner–actor simple IE	−0.002	(−0.007, 0.003)	0.506
	Direct effect	0.010	(−0.010, 0.030)	0.317
Husband	Total effect	0.012	(−0.009, 0.033)	0.248
	Indirect effect	0.000	(−0.005, 0.006)	0.833
	Actor–partner simple IE	0.002	(−0.001, 0.006)	0.139
	Partner–actor simple IE	−0.001	(−0.007, 0.003)	0.514
	Direct effect	0.012	(−0.009, 0.032)	0.237

## Data Availability

The datasets used and/or analyzed during the current study are available from the corresponding author upon reasonable request.

## Supplementary Materials

The following supporting information can be downloaded at https://www.mdpi.com/article/10.3390/medicina60030488/s1. Inventory S1: Inventory of Interpersonal Problems (IIP-32); Inventory S2: Multidimensional Scale of Perceived Social Support; Inventory S3: Core Symptom Index.
