# Supplementary material for: Social Inhibition and Depressive Symptoms among Couples with Children with Autism Spectrum Disorder: The Mediating Role of Perceived Family Support
Source: Medicina (Kaunas). 2024 Mar 15;60(3):488. doi: 10.3390/medicina60030488 (PMC10972493; doi:10.3390/medicina60030488)
Supplement: Supplementary file 1 [file medicina-60-00488-s001.zip › 5-IIP.pdf]

2022, Vol. 22, No. 2
ISSN 1667-4545
Recuperado de https://revistas.unc.edu.ar/index.php/revaluar

Laboratorio de Evaluación Psicológica y Educativa
Facultad de Psicología - Universidad Nacional de Córdoba

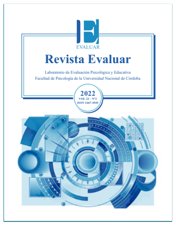

Inventory of Interpersonal Problems-32 (IIP-32): Psychometric properties and normative data in a clinical sample from Argentina

Inventario de Problemas Interpersonales-32 (IIP-32): Propiedades psicométricas y datos normativos en una muestra clínica de Argentina

Introduction
Methods
Results
Discussion
References

Juan Martín Gómez-Penedo [1, 2 *], Javier Fernández-Álvarez [3, 1], Mariana Maristany [3, 4], Agustín Freiberg-Hoffmann [1, 2]

1 - Facultad de Psicología, Universidad de Buenos Aires, Ciudad Autónoma de Buenos Aires, Argentina.
2 - Consejo Nacional de Investigaciones Científicas y Técnicas (CONICET), Ciudad Autónoma de Buenos Aires, Argentina.
3 - Asociación Aiglé, Valencia, Spain.
4 - Fundación Aiglé, Ciudad Autónoma de Buenos Aires, Argentina.

Recibido: 30/05/2022 **Revisado:** 07/06/2022 **Aceptado:** 20/06/2022

Abstract

This study analyzes the psychometric properties of the Inventory of Interpersonal Problems-32 (IIP-32), the most widely used instrument to measure relational difficulties. A sample of 2128 participants completed the IIP-32 and two additional measures of interpersonal difficulties before starting a psychotherapy treatment. To evaluate reliability, we analyzed internal consistency and item homogeneity. We analyzed the construct validity of IIP-32 through a confirmatory factor analysis and the concurrent validity through correlations between the IIP-32 and other measures of interpersonal deficits. The results of the study show excellent internal consistency and homogeneity of the items in the IIP-32. Furthermore, the results show construct validity as well as concurrent validity of the instrument. In sum, the results of this paper rank the IIP-32 as a reliable and valid instrument with important clinical implications to measure interpersonal difficulties in Argentina.

Keywords: *Inventory of Interpersonal Problems, IIP-32, validity, reliability, Argentina*

Resumen

Este estudio analiza las propiedades psicométricas del Inventario de Problemas Interpersonales-32 (IIP-32), la medida más utilizada para evaluar dificultades en las relaciones interpersonales. Una muestra de 2128 participantes completaron el IIP-32 y otras medidas de dificultades interpersonales antes de comenzar un tratamiento psicoterapéutico. Para evaluar la confiabilidad se analizaron medidas de consistencia interna y de homogeneidad de ítems. Se analizó la validez del constructo mediante un análisis factorial confirmatorio y la validez concurrente mediante correlaciones entre el IIP-32 y otras medidas de problemáticas interpersonales. Los resultados muestran excelentes niveles de consistencia interna y homogeneidad de ítems. A su vez, los resultados muestran la validez de constructo y validez de concurrente del instrumento. En síntesis, los resultados del trabajo posicionan al IIP-32 como un instrumento de confiabilidad y validez para estudiar dificultades interpersonales con importantes implicancias para la práctica clínica en Argentina.

Palabras clave: *Inventario de Problemas Interpersonales, IIP-32, validez, confiabilidad, Argentina*

***Correspondence to:** Juan Martín Gómez Penedo. Lavalle 2353, Buenos Aires, Argentina. Telephone: +54 9 11 6525 7053. E-mail: jmgomezpenedo@gmail.com

How to cite: Gómez-Penedo, J. M., Fernández-Álvarez, J., Maristany, M., & Freiberg-Hoffmann, A. (2022). Inventory of Interpersonal Problems-32 (IIP-32): Psychometric properties and normative data in a clinical sample from Argentina. *Revista Evaluar, 22*(2), 64-75. Retrieved from https://revistas.unc.edu.ar/index.php/revaluar

Participaron en la edición de este artículo: Melani Hajduczyk, María Eugenia Maiorana, Eugenia Barrionuevo, Pablo Carpintero, Stefano Macri, Florencia Ruiz, Mónica Serppe, Ricardo Hernández, Alicia Molinari.

Introduction

Interpersonal relationships have a fundamental relevance in people's daily lives (Horowitz, 2004; Lieberman, 2013; Wilson, 2012). Relational problems have been found to be associated with higher levels of psychopathology (Luo, Nuttall, Locke, & Hopwood, 2018; Segrin, 2001) and lower levels of life satisfaction (Froh et al., 2007). Meta-analytic studies have even suggested that difficulties in interpersonal relationships are a robust predictor of mortality comparable to well-established factors such as smoking and obesity (Holt-Lunstad, Smith, & Layton, 2010). For this reason, it is important to have valid and reliable instruments that enable an assessment of the degrees and nature of patients' interpersonal difficulties.

Considering that psychotherapy is by definition an interpersonal practice (Wampold & Imel, 2015), the relevance of instruments of this nature is particularly higher in the specific field of psychotherapy practice and research. On one hand, interpersonal problems represent one of the main reasons for consultation among people seeking treatment (Horowitz, 2004). On the other hand, relational difficulties have been identified as a dimension of change when evaluating the results of psychotherapy (Berghout, Zevalkink, Katzko, & de Jong, 2012; Salzer, Pincus, Winkelbach, Leichsenring, & Leibing, 2011). In other words, psychotherapy is expected to improve the way people relate to others. Finally, regarding the type of interpersonal problems that a person has, they can provide relevant information to determine the type of therapeutic process or to approach what it would be more beneficial for each patient (Gómez-Penedo, Constantino, Coyne, Westra, & Antony, 2017; Gómez-Penedo et al., 2020; Newman, Jacobson, Erickson, & Fisher, 2017; Zilcha-Mano, Muran, Eubanks, Safran, & Winston, 2018). This provides evidence in favor of the personalization of treatments and precision in mental health (Lutz, Schwartz, Gómez-Penedo, Boyle, & Deisenhofer, 2020; Zilcha-Mano, 2021).

Although there are different instruments to assess relational difficulties, the most widely used internationally is the Inventory of Interpersonal Problems (IIP; Horowitz, Rosenberg, Baer, Ureño, & Villaseñor, 1988). The IIP is considered a useful dimensional instrument to measure the interpersonal problems of patients at the beginning of the therapy and to assess its results (Maristany, 2008). The first version of this instrument had 127 items distributed in six subscales (i.e., *assertive*, *sociable*, *intimate*, *submissive*, *responsible* and *controlling*). Later, Horowitz et al. (2000) conducted a revised version of the IIP, which included 64 items (IIP-64) divided equally into eight subscales (*domineering*, *intrusive*, *overly nurturing*, *exploitable*, *nonassertive*, *socially inhibited*, *cold*, and *vindictive*). The subscales of this revised version configured octants distributed in a circumplex model consistent with classical interpersonal theories (Leary, 1957; Sullivan, 1955). In this way, the eight typologies of interpersonal problems are distributed around the two basic interpersonal dimensions of *agency* and *communion* (see Figure 1). The *communion* dimension describes the extent to which a person needs to establish close relationships with others (having issues of coldness/indifference at the negative pole and issues of being too dependent at the positive pole). The *agency* dimension describes the extent to which a person needs to influence other people or be influenced by others (having issues of submission at the negative pole and issues of being too dominant at the positive pole).

The IIP-64 has been widely used in clinical research and has been translated and adapted to different languages and cultures (Stiles & Hoglend, 1994; Thomas, Brähler & Strauß, 2011;

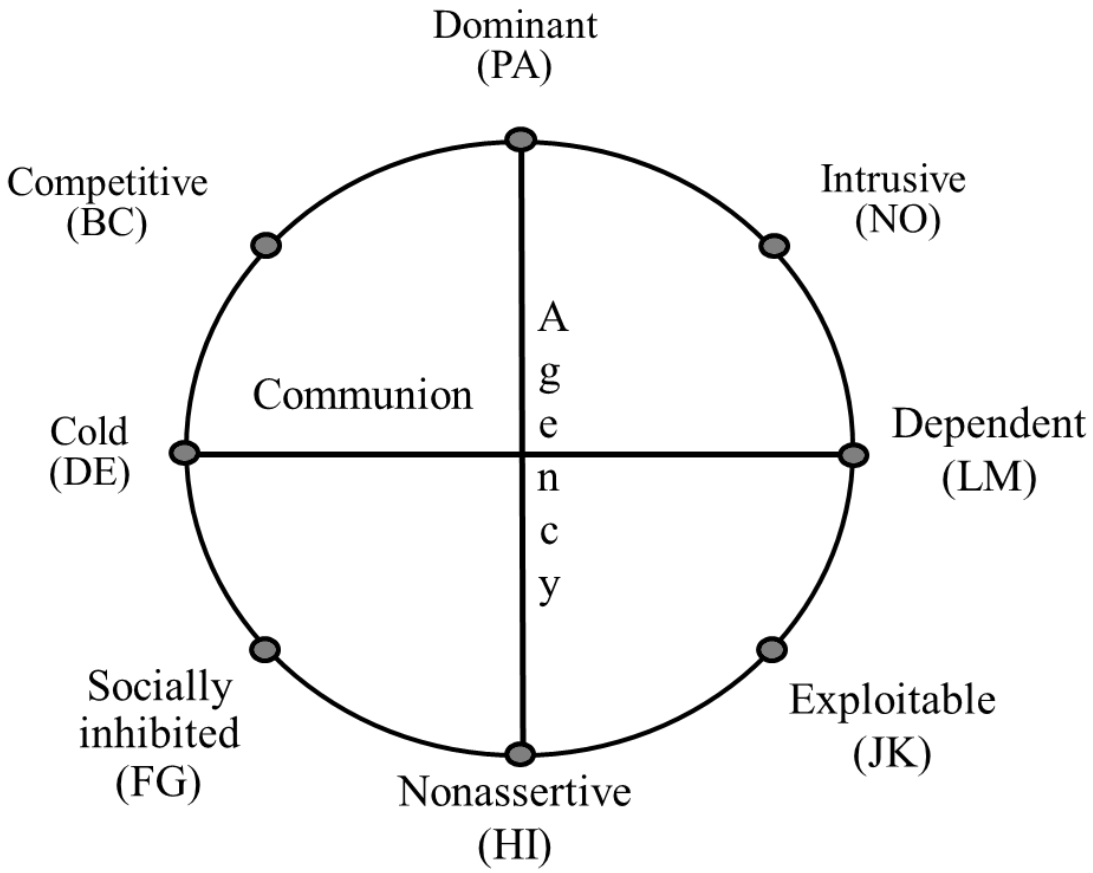

Figure 1. Subscales and dimensions of the circumplex model of the Inventory of Interpersonal Problems (Horowitz et al., 2000).

Vanheule, Desmet, & Rosseel, 2006) including the Argentinian context, where it showed adequate psychometric properties (Maristany, 2005). Despite the spread of the IIP-64 internationally, the number of items in the instrument remained a limitation for its use in clinical practice due to the burden it represented for patients. As a consequence, a shorter version of the IIP with 32 items was created (IIP-32; Barkham, Hardy, & Startup, 1996). This version can be considered ideal in clinical practice because it is brief and preserves the nature of the IIP-64 (McEvoy, Burgess, Page, Nathan, & Fursland, 2013). The IIP-32 has been adapted to countries such as Germany (Thomas et al., 2011), Norway (Vanheule et al., 2006), Italy (Lo Coco et al., 2018), Spain (Salazar, Martí, Soriano, Beltram, & Adam, 2010) and Portugal (Faustino & Vasco, 2020), among others. However, in the literature analyzed, we could not find any study that analyzed the psychometric properties of the IIP-32 in the Latin American context.

In this framework, the aim of this paper is to analyze the psychometric properties of the IIP-32 in a clinical sample from Argentina. In particular, the levels of reliability (internal consistency and item homogeneity) and validity (construct validity and concurrent validity) of the inventory are analyzed. An instrument with adequate levels of reliability and validity would be a relevant contribution to both research and clinical practice in mental health.

Methods

Participants

The sample consisted of 2128 participants who completed the IIP-32 during the years 2009 and 2021. The mean age of the participants was 33.7 years (SD = 12.42). 62% of the participants were women and 38% were men. There was no participant diagnostic data or other clinical data.

Instruments

Inventory of Interpersonal Problems (IIP-32; Horowitz, 2000). This scale is a brief 32-item version of the Interpersonal Problems Inventory (Horowitz et al., 1988), which assesses people's difficulties in their relationships with others. The subjects of study are presented with a list of problems that people tend to have when interacting with others, being either excessive behaviors or inhibitions. Each subject has to assess whether each item represents a problem when interacting with a significant person in their life in the last two weeks. The answers run on a scale from 0 (*Not at all*) to 4 (*Very much*). The 32 items are distributed in eight subscales of interpersonal problems: *domineering*, *intrusive*, *overly nurturing*, *exploitable*, *nonassertive*, *socially inhibited*, *cold* and *vindictive*. Some of the items are: *It is difficult for me to say no to other people* or *It is difficult for me to experience a feeling of love for another person*. In addition to the subscales, the instrument helps to compute a total score of interpersonal distress. Additionally, it can calculate the two interpersonal dimensions of *agency* and *communion*, based on weighted combinations of the eight subscales. For the calculation of *agency* = .25 (domineering - nonassertive + .71[intrusive + vindictive - socially inhibited - exploitable]); *communion* = .25 (overly nurturant - cold + .71[intrusive - vindictive - socially inhibited + exploitable]; Ruiz et al., 2004). The IIP-64 has a Spanish adaptation in Argentina with excellent psychometric properties (Maristany, 2005).

Symptom Checklist (SCL-90; Derogatis, 1994) [Spanish version; Casullo & Pérez, 2008]. This 90-item self-report inventory measures the presence and severity of psychiatric symptomatology. The instrument has nine primary dimensions and three global indexes that represent the levels of psychological distress. Some dimensions, for example, include symptoms such as *anxious and depressive* or *psychotic and somatic*. Each item is answered on a five-point Likert-type scale ranging from 0 (*Not at all*) to 4 (*Extremely*). Especially important for this study is the fact that the SCL-90 includes an interpersonal sensitivity dimension (e.g., *Feeling critical of others* or *Feeling shy or uneasy with the opposite sex*). The SCL-90 has been adapted in Argentina and has demonstrated good levels of internal consistency for all scales and for the general index (Casullo & Pérez, 2008).

Outcome Questionnaire (OQ.45; Lambert et al., 1996)[Spanish version; von Bergen, 2002]. The OQ.45 is a 45-item self-report questionnaire that measures progress and results in psychotherapy and monitors the evolution of patients with successive measurements. The items are grouped into three subscales that describe the periodicity in experiences of distress in patients in different areas: 1) symptomatic distress, 2) interpersonal relations and 3) social role. The items are rated on a five-point Likert-type scale ranging from 1 = *Never* to 5 = *Almost always*. In this study, the subscale of interpersonal relations was used, which include items such as *I get along well with others*. The Spanish version of the instrument has shown adequate psychometric properties in the Argen-

tine context (Fernández-Álvarez, Hirsh, Maristany, & Torrente, 2005).

Procedures

Patients attending a private clinic for psychotherapeutic treatment completed the IIP-32 as part of the diagnostic and clinical assessment process. In addition, prior to their treatment, the patients completed the SCL-90 interpersonal sensitivity subscale and the OQ.45 interpersonal relations subscale. All participants signed a written consent under a confidentiality clause for the use of these data for research purposes.

Data analysis

For the data analysis, LISREL 8.8 software (Jöreskog & Sörbom, 1993) and open-source software R (R Core Team, 2021), particularly with the Hmisc (Harrell Jr, 2021) and psych (Revelle, 2017) packages, were used. Internal consistency and item homogeneity were analyzed as reliability measures of the IIP-32. The internal consistency was established by Cronbach's alpha. Since the instrument items have an ordinal measurement scale and a five-point response scale, in addition to Cronbach's alpha, the ordinal alpha is presented (Freiberg-Hoffmann, Stover, de la Iglesia, & Fernández-Liporace, 2013; Gadermann, Guhn, & Zumbo, 2012). Values between .70 and .90 are considered acceptable (Tavakol & Dennick, 2011). To analyze item homogeneity, we calculated corrected item-scale correlations. Correlations in the range from .30 to .80 are indicators of adequate levels of item homogeneity (Rattray & Jones, 2007).

To measure the validity of the instrument, construct and concurrent validity were evaluated. For the construct validity, a confirmatory factor analysis was performed followed by an analysis of factorial invariance. For this purpose, the diagonally weighted least squares (DWLS) estimation method was employed, an alternative to the maximum likelihood method for ordinal items and large sample sizes (Kiliç & Doğan, 2021; Li, 2016; Mîndrilă, 2010). To interpret the model fit, comparative fit index (CFI), Tucker-Lewis index (TLI) and root mean square error of approximation (RMSEA) were used. Values above .90 in CFI and TLI (Schumacker & Lomax, 2016) and below .08 in RMSEA (Schumacker & Lomax, 2016) were indicators of an adequate fit of the model. Due to the nature of the instrument, a bifactor model was adjusted (Reise, 2012). Additionally, the bias of the bifactor model items in the estimation of unidimensional measures was evaluated through the percent of uncontaminated correlations (PUC) and average relative parameter bias (ARPB) statistics whose values above .80 and below 15% respectively were indicators of low bias (Davidov, Billiet, Meuleman, & Schmidt, 2018; Hammer, 2016; Rodriguez, Reise, & Haviland, 2016).

The analysis of factorial invariance was performed by segmenting the sample according to sex variable —men and women—. Three nested models with different levels of restriction (configural, metric, scalar) were analyzed. The invariance was interpreted with the CFI and RMSEA indexes whose differences should be less than .01 and .015 respectively (Putnick & Bornstein, 2016; Rial-Boubeta, Varela-Mallou, Abalo-Piñeiro, & Levy-Mangin, 2006).

For the concurrent validity study, Pearson product-moment correlations were calculated between the IIP-32 total score, the SCL-90 interpersonal sensitivity scale and the OQ.45 interpersonal relationship distress scale. For evidence of adequate concurrent validity, correlation values in the range of .50 to .85 are expected (Rial-Boubeta

et al., 2006).

The code of the study analyses and its results will be published on the open science framework page (after the anonymous peer review process), in rmarkdown format (Allaire et al., 2022).

Results

Descriptive analysis (Sample 1)

Table 1 presents the descriptive analyses of all the items of the IIP-32. Table 2 reports descriptive analyses of the scales and the total score of the IIP-32.

Table 1
Descriptive analysis and corrected item-scale correlations of the IIP-32 items.

Item	Mean	SD	Scale	*r*
Item 1	2.09	1.29	JK	.49
Item 2	1.48	1.37	FG	.72
Item 3	1.07	1.17	NO	.39
Item 4	1.32	1.33	HI	.51
Item 5	1.39	1.36	FG	.66
Item 6	1.41	1.30	HI	.49
Item 7	1.29	1.16	HI	.53
Item 8	1.36	1.28	JK	.40
Item 9	1.17	1.31	FG	.70
Item 10	1.17	1.27	DE	.63
Item 11	0.76	1.07	DE	.54
Item 12	1.61	1.30	HI	.53
Item 13	1.05	1.31	DE	.56
Item 14	0.60	1.03	BC	.59
Item 15	1.06	1.18	DE	.58
Item 16	0.89	1.15	BC	.61
Item 17	1.27	1.30	BC	.41
Item 18	0.67	1.10	BC	.56
Item 19	1.23	1.32	FG	.52
Item 20	2.01	1.26	JK	.36
Item 21	1.54	1.32	NO	.49
Item 22	0.80	1.07	PA	.52
Item 23	1.77	1.30	LM	.53
Item 24	0.82	1.09	NO	.36
Item 25	1.08	1.19	PA	.49
Item 26	1.66	1.30	LM	.60
Item 27	1.58	1.25	LM	.57
Item 28	1.00	1.17	PA	.38
Item 29	1.05	1.16	NO	.57
Item 30	0.94	1.10	PA	.68
Item 31	0.78	1.05	JK	.40
Item 32	1.92	1.27	LM	.41

Note. SD = Standard deviation. For scale references see Figure 1.

Table 2
Descriptive analyses of the IIP-32 total score and scales.

Scales	Mean	SD	Range
PA	0.94	0.81	[0; 4]
BC	0.88	0.87	[0; 4]
DE	1.02	0.94	[0; 4]
FG	1.29	1.08	[0; 4]
HI	1.40	0.94	[0; 4]
JK	1.54	0.84	[0; 4]
LM	1.73	0.95	[0; 4]
NO	1.11	0.84	[0; 4]
Total	1.24	0.56	[0; 3.31]

Table 3
Cronbach's alpha and ordinal alpha for IIP-32 scales.

Scales	Cronbach's alpha	Ordinal alpha
PA	.70	.76
BC	.74	.81
DE	.77	.83
FG	.82	.86
HI	.73	.77
JK	.63	.68
LM	.73	.77
NO	.67	.72
TOTAL	.87	.97

Reliability

Internal consistency. The items of the IIP-32 presented a Cronbach's alpha of .88. Due to the ordinal level of measurement of the items, the ordinal alpha of the IIP-32 items was computed with a value of .90. Table 3 presents the Cronbach's alphas and ordinal alphas at the level of the eight scales of the IIP-32.

Item homogeneity. Table 1 shows the item-scale correlations. None of the items of the IIP-32 presented correlations lower than .30 or higher than .80 with the items of its scale. The items with greatest item-total adjusted correlations of each scale were the following: item 30 (PA, r = .68), item 14 (BC, r = .59), item 10 (SD, r = .63), item

Table 4
Parameters and coefficients of determination of the confirmatory factor model.

Scale - Items	λ Subscale	Scale - Items	λ Total	R^2
PA – Item 22	.732	T – Item 22	.301	.626
PA – Item 25	.511	T – Item 25	.313	.359
PA – Item 28	.370	T – Item 28	.305	.230
PA – Item 30	.730	T – Item 30	.320	.635
BC – Item 14	.612	T – Item 14	.595	.650
BC – Item 16	.603	T – Item 16	.491	.605
BC – Item 17	.509	T – Item 17	.196	.297
BC – Item 18	.543	T – Item 18	.530	.575
DE – Item 10	.776	T – Item 10	.481	.833
DE – Item 11	.222	T – Item 11	.719	.567
DE – Item 13	.477	T – Item 13	.497	.475
DE – Item 15	.264	T – Item 15	.702	.562
FG – Item 2	.665	T – Item 2	.566	.762
FG – Item 5	.603	T – Item 5	.535	.650
FG – Item 9	.601	T – Item 9	.616	.741
FG – Item 19	.259	T – Item 19	.651	.491
HI – Item 4	.363	T – Item 4	.604	.497
HI – Item 6	.212	T – Item 6	.602	.407
HI – Item 7	.269	T – Item 7	.610	.445
HI – Item 12	.395	T – Item 12	.581	.493
JK – Item 1	.588	T – Item 1	.483	.579
JK – Item 8	.275	T – Item 8	.511	.337
JK – Item 20	.355	T – Item 20	.306	.220
JK – Item 31	.280	T – Item 31	.598	.436
LM – Item 23	.592	T – Item 23	.394	.506
LM – Item 26	.691	T – Item 26	.308	.572
LM – Item 27	.683	T – Item 27	.267	.537
LM – Item 32	.448	T – Item 32	.240	.259
NO – Item 3	.435	T – Item 3	.421	.366
NO – Item 21	.733	T – Item 21	.084	.545
NO – Item 24	.408	T – Item 24	.284	.247
NO – Item 29	.745	T – Item 29	.298	.645

2 (FG, r = .72), item 7 (HI, r = .53), item 1 (JK, r = .49), item 26 (LM, r = .60) and item 29 (NO, r = .59). The average of these eight aggregated items correlated strongly with the total IIP-32 score (r = .91).

Validity

Construct validity. A bifactor model was tested. Table 4 shows the parameters of the confirmatory factor model and the coefficient of determination for each item. The model verified an adequate fit to the empirical data with CFI indices of .93, TLI of .92 and RMSEA [CI90%] of .076 [.074, .078]. Acceptable PUC indices of .90 % and ARPB of 12% were obtained.

The factorial invariance of the bifactor model was tested by segmenting the sample according to sex. The metric equivalence of the model between men and women was verified (Table 5).

Table 5
Factorial invariance of the bifactor model segmenting the sample by sex.

	CFI	Δ CFI	RMSEA	Δ RMSEA
Configural	.941	-	.075 [.073, .077]	-
Metric	.938	.003	.074 [.072, .076]	.001
Scalar	.935	.006	.075 [.073, .077]	0

Concurrent validity. Pearson correlations showed significant direct associations of the IIP-32 with the OQ.45 interpersonal relationship distress scale ($r = .59$, $p < .001$) and with the SCL-90 interpersonal sensitivity scale ($r = .62$, $p < .001$). In both cases the correlations demonstrated a large effect size in the associations of the IIP-32 and the other measures of interpersonal distress (Dominguez-Lara, 2018).

Discussion

The aim of this study was to analyze the psychometric properties of the IIP-32 in a sample of patients from Argentina. For this purpose, these patients completed a version of the IIP in the diagnostic evaluation process, prior to initiating psychotherapeutic treatment in a private clinical center. The results of this study show adequate levels of reliability and validity of the instrument in the Argentinian context.

Internal consistency and item homogeneity were used to evaluate reliability. The instrument showed an excellent level of internal consistency in its total score, with a Cronbach's alpha of .88 and an ordinal alpha of .90 and adequate levels of internal consistency in six of the eight dimensions, with alphas in the range of .70 to .90 (Tavakol & Dennick, 2011). The *intrusive* (NO; alpha = .67) and *exploitable* (JK; alpha = .63) scales presented alphas below the expected range.

In addition, adequate levels of item homogeneity were observed in the instrument, with all items presenting corrected item-scale correlations in the suggested range of .30 to .80 (Rattray & Jones, 2007). The fact that the correlations are greater than .30 implies that there is some degree of minimal association between the items, while correlations below .80 suggest that the items are not redundant.

Both construct and concurrent validity of the IIP-32 were analyzed. The confirmatory factor analysis demonstrated adequate construct validity of the IIP-32 in the Argentine context, with all goodness-of-fit measures within the parameters suggested in the literature. The factor model obtained CFI (.93) and TLI (.92), values above .90 (Schumacker & Lomax, 2016), while the RMSEA index (.07) was below .08 (Schumacker & Lomax, 2016). Acceptable PIC and ARPB values were also obtained, which indicated low item bias in the bifactor model (Davidov et al., 2018). The analysis of factorial invariance verified that the structure of the instrument remains metrically equivalent in both male and female samples. In the analysis of the items, 17 of the 32 items contributed at least 50% of their variability to the explanation of the latent variables of the model (Kline, 2005). This would indicate that most of the items that make up the model have a high degree of representativeness to assess the construct of interpersonal problems.

Pearson's correlations between the IIP-32 total score and the subscales of interpersonal relationship distress of the OQ.45 ($r = .59$) and interpersonal sensitivity of the SCL-90 ($r = .62$) demonstrated concurrent validity between IIP-32 scores and instruments that measure close constructs. By being in the range of .50 to .85, the correlations indicate that the constructs are associated, but not identical (Rial-Boubeta et al., 2006).

In summary, the results of this work present the IIP-32 as a reliable and valid instrument for the evaluation of interpersonal problems in patients at the beginning of their psychotherapy treatment. The IIP-32 encourages a global evaluation of the degree of difficulty that people have in establishing and maintaining relationships and also helps to perform a thorough analysis of the type of difficulties that an individual may have in

a relationship. The characterization of these difficulties has great clinical relevance when evaluating patients, designing treatments and analyzing their results. Unlike the original IIP (127 items) and the IIP-64 (64 items), the IIP-32 is a shorter version that involves less time for patients to complete. Therefore, the use of the IIP-32 would improve the measurements of interpersonal problems in clinical practice and its use for research (Gómez-Penedo et al., 2021).

It should be noted that this study has a number of limitations that will need to be addressed in future research. First, the instruments used in the validation of the IIP-32 were entirely self-report measures. Consequently, the correlations between measurements increase because they come from the same source. Future research should include measures that are completed by other sources (outside observers, therapists, acquaintances, etc.) to explore the concurrent validity of the IIP-32. These measures would provide detailed and complementary information about the interpersonal problems of the participants, providing valuable information in the study of the subject. Second, as it was previously mentioned, there was no information on the diagnosis of the participants. This limits the type of analyses used, since the results obtained cannot be compared according to the diagnosis of the participants. For example, significant differences could be found depending on whether patients are diagnosed with personality disorders or depressive disorders. For this reason, future research could be favored by incorporating, through a unified diagnostic criterion, systematized information about the diagnosis of the participants. It should be noted as well that in this study the sample used was made up of participants from a clinical population, which affects the generalization of the results. Therefore, a future investigation should include participants from both a clinical and non-clinical population. Therefore, evaluation procedures could be used to analyze differences between groups. Finally, no repeated measures of the instrument were taken. Future research could study the instrument's sensitivity to change as well as detect and create criteria for clinically significant change that could be used in monitoring and feedback systems for therapists.

The results of this study place the IIP-32 as a reliable and valid instrument for the Argentinian context, therefore, it is a relevant resource for clinical studies and research in psychotherapy.

References

Allaire, J., Xie, Y., McPherson, J., Luraschi, J., Ushey, K., Atkins, A., Wickham, H., Cheng, J., Chang, W., & Iannone, R. (2022). rmarkdown: Dynamic documents for R. R package version 2.14. Recuperado de https://github.com/rstudio/rmarkdown

Barkham, M., Hardy, G. E., & Startup, M. (1996). The IIP-32: A short version of the Inventory of Interpersonal Problems. *British Journal of Clinical Psychology, 35*(1), 21-35. doi: 10.1111/j.2044-8260.1996.tb01159.x

Berghout, C. C., Zevalkink, J., Katzko, M. W., & de Jong, J. T. (2012). Changes in symptoms and interpersonal problems during the first 2 years of long-term psychoanalytic psychotherapy and psychoanalysis. *Psychology and Psychotherapy: Theory, Research and Practice, 85*(2), 203-219. doi: 10.1111/j.2044-8341.2011.02022.x

Casullo, M., & Pérez, M. (2008). *El inventario de síntomas SCL-90-R de L. Derogatis.* Universidad de Buenos Aires. Recuperado de https://www.psi.uba.ar

Davidov, E., Billiet, J., Meuleman, B., & Schmidt, P. (2018). *Cross-Cultural Analysis: Methods and Applications (2nd ed.)*. Routledge. doi: 10.4324/9781315537078

Derogatis, L. (1994). *SCL-90-R.* Minneapolis, MN: NCS.

Dominguez-Lara, S. (2018). Magnitud del efecto, una guía

rápida. *Educación Médica, 19*(4), 251-254. doi: 10.1016/j.edumed.2017.07.002

Faustino, B., & Vasco, A. B. (2020). Factor structure and convergent validity of the Portuguese version of the Inventory of Interpersonal Problems-32. *Journal of Relationships Research, 11*. doi: 10.1017/jrr.2020.18

Fernández-Álvarez, H., Hirsh, H., Maristany, M., & Torrente, F. (2005). Propiedades psicométricas del OQ-45.2 en la Argentina: Un estudio piloto. Poster presentado en 4° Congreso Mundial de Psicoterapia, Buenos Aires, Argentina.

Freiberg-Hoffmann, A., Stover, J. B., De la Iglesia, G., & Fernández Liporace, M. (2013). Correlaciones policóricas y tetracóricas en estudios factoriales exploratorios y confirmatorios. *Ciencias Psicológicas, 7*(2), 151-164. doi: 10.22235/cp.v7i1.1057

Froh, J. J., Fives, C. J., Fuller, J. R., Jacofsky, M. D., Terjesen, M. D., & Yurkewicz, C. (2007). Interpersonal relationships and irrationality as predictors of life satisfaction. *The Journal of Positive Psychology, 2*(1), 29-39. doi: 10.1080/17439760601069051

Gadermann, A. M., Guhn, M., & Zumbo, B. D. (2012). Estimating ordinal reliability for Likert-type and ordinal item response data: A conceptual, empirical, and practical guide. *Practical Assessment, Research, and Evaluation, 17*(1), 3-13. doi: 10.7275/n560-j767

Gómez-Penedo, J. M., Areas, M. A., Manubens, R., Babl, A. M., Challú, L., Juan, S., … Grosse-Holtforth, M. (2021). Propiedades psicométricas del Hopkins Symptom Checklist (HSCL-11) en Argentina: Un instrumento para monitoreo y feedback en psicoterapia. *Revista Evaluar, 21*(2), 33-47. doi: 10.35670/1667-4545.v21.n2.34393

Gómez-Penedo, J. M., Babl, A., Krieger, T., Heinonen, E., & Flückiger, C. (2020). Interpersonal agency as predictor of the within-patient alliance effects on depression severity. *Journal of Consulting and Clinical Psychology, 88*(4), 338-349. doi: 10.1037/ccp0000475

Gómez-Penedo, J. M., Constantino, M. J., Coyne, A. E., Westra, H. A., & Antony, M. M. (2017). Markers for context-responsiveness: Client baseline interpersonal problems moderate the efficacy of two psychotherapies for generalized anxiety disorder. *Journal of Consulting and Clinical Psychology, 85*(10), 1000-1011. doi: 10.1037/ccp0000233

Hammer, J. H. (2016). Percent of Uncontaminated Correlations (PUC) Calculator: A Microsoft Excel-based tool to calculate the PUC statistic. Recuperado de http://drjosephhammer.com

Harrell Jr, F. (2021). Hmisc: Harrell miscellaneous. R package version 4.6-0. Recuperado de https://cran.r-project.org

Holt-Lunstad, J., Smith, T. B., & Layton, J. B. (2010). Social relationships and mortality risk: A meta-analytic review. *PLoS Medicine, 7*(7), e1000316. Recuperado de https://journals.plos.org

Horowitz, L. M. (2004). *Interpersonal foundations of psychopathology.* American Psychological Association. Recuperado de https://www.apa.org

Horowitz, L. M., Alden, L. E., Wiggins, J. S., & Pincus, A. L. (2000). *IIP, Inventory of Interpersonal Problems Manual.* San Antonio: The Psychological Corporation. Recuperado de https://fetzer.org

Horowitz, L. M., Rosenberg, S. E., Baer, B. A., Ureño, G., & Villaseñor, V. S. (1988). Inventory of Interpersonal Problems: Psychometric properties and clinical applications. *Journal of Consulting and Clinical Psychology, 56*(6), 885-892. doi: 10.1037//0022-006x.56.6.885

Jöreskog, K., & Sörbom, D. (1993). LISREL 8 [Computer software]. Chicago, IL: Scientific Software International, Inc. Recuperado de https://ssicentral.com

Kiliç, A. F., & Doğan, N. (2021). Comparison of confirmatory factor analysis estimation methods on mixed-format data. *International Journal of Assessment Tools in Education, 8*(1), 21-37. Recuperado de https://files.eric.ed.gov

Kline, R. (2005). *Principles and Practice of Structural equation modeling (2nd ed.)*. Guilford Press.

Lambert, M. J., Burlingame, G. M., Umphress, V., Hansen, N. B., Vermeersch, D. A., Clouse, G. C., & Yanchar,

S. C. (1996). Reliability and validity of the Outcome Questionnaire. *Clinical Psychology & Psychotherapy: An International Journal of Theory and Practice, 3*(4), 249-258. doi: 10.1002/(SICI)1099-0879(199612)3:4<249::AID-CPP106>3.0.CO;2-S

Leary, T. (1957). *Interpersonal diagnosis of personality; a functional theory and methodology for personality evaluation.* Ronald Press.

Li, C. H. (2016). Confirmatory factor analysis with ordinal data: Comparing robust maximum likelihood and diagonally weighted least squares. *Behavior Research Methods, 48*(3), 936-949. doi: 10.3758/s13428-015-0619-7

Lieberman, M. D. (2013). *Social: Why our brains are wired to connect.* OUP Oxford.

Lo Coco, G., Mannino, G., Salerno, L., Oieni, V., Di Fratello, C., Profita, G., & Gullo, S. (2018). The Italian version of the inventory of interpersonal problems (IIP-32): Psychometric properties and factor structure in clinical and non-clinical groups. *Frontiers in Psychology, 9*, 341. doi: 10.3389/fpsyg.2018.00341

Luo, X., Nuttall, A. K., Locke, K. D., & Hopwood, C. J. (2018). Dynamic longitudinal relations between binge eating symptoms and severity and style of interpersonal problems. *Journal of Abnormal Psychology, 127*(1), 30-42. doi: 10.1037/abn0000321

Lutz, W., Schwartz, B., Gómez-Penedo, J. M., Boyle, K., & Deisenhofer, A. K. (2020). Working towards the development and implementation of precision mental healthcare: An example. *Administration and Policy in Mental Health and Mental Health Services Research, 47*(5), 856-861. doi: 10.1007/s10488-020-01053-y

Maristany, M. (2005). *Problemas en las relaciones interpersonales y trastornos de la personalidad.* Universidad de Belgrano. Recuperado de http://repositorio.ub.edu.ar

Maristany, M. (2008). Diagnóstico y evaluación de las relaciones interpersonales y sus perturbaciones. *Revista Argentina de Clínica Psicológica, 17*(1), 19-36. Recuperado de http://www.redalyc.org/articulo.oa?id=281921796002

McEvoy, P. M., Burgess, M. M., Page, A. C., Nathan, P., & Fursland, A. (2013). Interpersonal problems across anxiety, depression, and eating disorders: A transdiagnostic examination. *British Journal of Clinical Psychology, 52*(2), 129-147. doi: 10.1111/bjc.12005

Mîndrilă, D. (2010). Maximum likelihood (ML) and diagonally weighted least squares (DWLS) estimation procedures: A comparison of estimation bias with ordinal and multivariate non-normal data. *International Journal of Digital Society, 1*(1), 60-66. Recuperado de https://infonomics-society.org

Newman, M. G., Jacobson, N. C., Erickson, T. M., & Fisher, A. J. (2017). Interpersonal problems predict differential response to cognitive versus behavioral treatment in a randomized controlled trial. *Behavior Therapy, 48*(1), 56-68. doi: 10.1016/j.beth.2016.05.005

Putnick, D. L., & Bornstein, M. H. (2016). Measurement invariance conventions and reporting: The state of the art and future directions for psychological research. *Developmental Review, 41*, 71-90. doi: 10.1016/j.dr.2016.06.004

R Core Team. (2021). R: A language and environment for statistical computing. R Foundation for Statistical Computing. Recuperado de https://www.r-project.org

Rattray, J., & Jones, M. C. (2007). Essential elements of questionnaire design and development. *Journal of Clinical Nursing, 16*(2), 234-243. doi: 10.1111/j.1365-2702.2006.01573.x

Reise, S. P. (2012). The rediscovery of bifactor measurement models. *Multivariate Behavioral Research, 47*(5), 667-696. doi: 10.1080/00273171.2012.715555

Revelle, W. R. (2017). psych: Procedures for personality and psychological research. Northwestern University. Recuperado de https://www.scholars.northwestern.edu

Rial-Boubeta, A., Varela-Mallou, J., Abalo-Piñeiro, J., & Levy-Mangin, J. (2006). El análisis factorial confirmatorio. En J. P. Lévy Mangin (Dir.) & J. Varela Mallou (Coord.), *Modelización con estructuras de*

covarianzas en ciencias sociales: temas esenciales, avanzados y aportaciones especiales (pp. 119-154). Netbiblo, S. L.

Rodriguez, A., Reise, S. P., & Haviland, M. G. (2016). Applying bifactor statistical indices in the evaluation of psychological measures. *Journal of Personality Assessment, 98*(3), 223-237. doi: 10.1080/00223891.2015.1089249

Ruiz, M. A., Pincus, A. L., Borkovec, T. D., Echemendia, R. J., Castonguay, L. G., & Ragusea, S. A. (2004). Validity of the Inventory of Interpersonal Problems for predicting treatment outcome: An investigation with the Pennsylvania Practice Research Network. *Journal of Personality Assessment, 83*(3), 213-222. doi: 10.1207/s15327752jpa8303_05

Salazar, J., Martí, V., Soriano, S., Beltran, M., & Adam, A. (2010). Validity of the Spanish version of the Inventory of Interpersonal Problems and its use for screening personality disorders in clinical practice. *Journal of Personality Disorders, 24*(4), 499-515. doi: 10.1521/pedi.2010.24.4.499

Salzer, S., Pincus, A. L., Winkelbach, C., Leichsenring, F., & Leibing, E. (2011). Interpersonal subtypes and change of interpersonal problems in the treatment of patients with generalized anxiety disorder: A pilot study. *Psychotherapy, 48*(3), 304-310. doi: 10.1037/a0022013

Schumacker, E., & Lomax, G. (2016). *A Beginner's Guide to Structural Equation Modelling (4th ed.)*. Routledge.

Segrin, C. (2001). *Interpersonal processes in psychological problems*. Guilford Press.

Stiles, T., & Hoglend, P. (1994). IIP-64-C - norsk oversettelse [IIP- 64-C - Norwegian translation].

Sullivan, H. (1955). *The interpersonal Theory of Psychiatry.* London: Tavistock.

Tavakol, M., & Dennick, R. (2011). Making sense of Cronbach's alpha. *International Journal of Medical Education, 2*, 53-55. doi: 10.5116/ijme.4dfb.8dfd

Thomas, A., Brähler, E., & Strauß, B. (2011). IIP-32: Entwicklung, validierung und normierung einer kurzform des inventars zur erfassung interpersonaler probleme. *Diagnostica, 57*(2), 68-83. doi: 10.1026/0012-1924/a000034

Vanheule, S., Desmet, M., & Rosseel, Y. (2006). The factorial structure of the Dutch translation of the inventory of interpersonal problems: a test of the long and short versions. *Psychological Assessment, 18*(1), 112-117. doi: 10.1037/1040-3590.18.1.112

Von Bergen, A., & de la Parra, G. (2002). OQ-45.2, Cuestionario para evaluación de resultados y evolución en psicoterapia: Adaptación, validación e indicaciones para su aplicación e interpretación. *Terapia Psicológica, 20*(2), 161-176. Recuperado de https://pesquisa.bvsalud.org/portal/resource/pt/lil-389263?lang=es

Wampold, B. E., & Imel, Z. E. (2015). *The great psychotherapy debate: The evidence for what makes psychotherapy work.* Routledge.

Wilson, E. O. (2012). *The social conquest of earth.* WW Norton & Company.

Zilcha-Mano, S. (2021). Toward personalized psychotherapy: The importance of the trait-like/state-like distinction for understanding therapeutic change. *American Psychologist, 76*(3), 516-528. doi: 10.1037/amp0000629

Zilcha-Mano, S., Muran, J. C., Eubanks, C. F., Safran, J. D., & Winston, A. (2018). Not just a non-specific factor: Moderators of the effect of within-and between-clients alliance on outcome in CBT. *Cognitive Therapy and Research, 42*(2), 146-158. doi: 10.1007/s10608-017-9866-5
