# Supplementary material for: Social Inhibition and Depressive Symptoms among Couples with Children with Autism Spectrum Disorder: The Mediating Role of Perceived Family Support
Source: Medicina (Kaunas). 2024 Mar 15;60(3):488. doi: 10.3390/medicina60030488 (PMC10972493; doi:10.3390/medicina60030488)
Supplement: Supplementary file 1 [file medicina-60-00488-s001.zip › CSI-15 Eng .pdf]

CSI-15

Core Symptom Index

**In the past 1 week** How often do you feel disturbed by the following symptoms<br>Please use X on the number you agree the most	Not at all	little	some what	Quite much	most
1. A ringing or Buzzing in the ear(s)	0	1	2	3	4
2. Suicidal idea	0	1	2	3	4
3. Palpitation	0	1	2	3	4
4. crying	0	1	2	3	4
5. self-blaming	0	1	2	3	4
6. feeling lonely	0	1	2	3	4
7. depressed	0	1	2	3	4
8. Trouble catching your breath	0	1	2	3	4
9. Hot or cold spells	0	1	2	3	4
10. Feeling numb or tinging	0	1	2	3	4
11. A fullness in head or nose	0	1	2	3	4
12. Discomfort when in the crowd	0	1	2	3	4
13. Upset when being left alone	0	1	2	3	4
14. Feeling agitated	0	1	2	3	4
15. feeling urge to do things	0	1	2	3	4

CSI-15

Wongpakaran N, Wongpakaran T, Lertkachatarn S, Sirirak T, Kuntawong P.Core Symptom Index (CSI): testing for bifactor model and differential item functioning. Int Psychogeriatr. 2019 Dec;31(12):1769-1779. doi: 10.1017/S1041610219000140. Epub 2019 Mar 27. PMID: 30915932
