# Supplementary material for: Social Inhibition and Depressive Symptoms among Couples with Children with Autism Spectrum Disorder: The Mediating Role of Perceived Family Support
Source: Medicina (Kaunas). 2024 Mar 15;60(3):488. doi: 10.3390/medicina60030488 (PMC10972493; doi:10.3390/medicina60030488)
Supplement: Supplementary file 1 [file medicina-60-00488-s001.zip › MSPSS.pdf]

ResearchGate

See discussions, stats, and author profiles for this publication at: https://www.researchgate.net/publication/311534896

Multidimensional Scale of Perceived Social Support (MSPSS) - Scale Items and Scoring Information

Research · December 2016

CITATIONS
90

READS
198,016

1 author:

Gregory Zimet
Indiana University School of Medicine
516 PUBLICATIONS **28,759** CITATIONS

SEE PROFILE

All content following this page was uploaded by Gregory Zimet on 09 December 2016.

The user has requested enhancement of the downloaded file.

Multidimensional Scale of Perceived Social Support

Instructions: We are interested in how you feel about the following statements. Read each statement carefully. Indicate how you feel about each statement.

Circle the “1” if you **Very Strongly Disagree**
Circle the “2” if you **Strongly Disagree**
Circle the “3” if you **Mildly Disagree**
Circle the “4” if you are **Neutral**
Circle the “5” if you **Mildly Agree**
Circle the “6” if you **Strongly Agree**
Circle the “7” if you **Very Strongly Agree**

	Very Strongly Disagree	Strongly Disagree	Mildly Disagree	Neutral	Mildly Agree	Strongly Agree	Very Strongly Agree
1. There is a special person who is around when I am in need.	1	2	3	4	5	6	7
2. There is a special person with whom I can share joys and sorrows.	1	2	3	4	5	6	7
3. My family really tries to help me.	1	2	3	4	5	6	7
4. I get the emotional help & support I need from my family.	1	2	3	4	5	6	7
5. I have a special person who is a real source of comfort to me.	1	2	3	4	5	6	7
6. My friends really try to help me.	1	2	3	4	5	6	7
7. I can count on my friends when things go wrong.	1	2	3	4	5	6	7
8. I can talk about my problems with my family.	1	2	3	4	5	6	7
9. I have friends with whom I can share my joys and sorrows.	1	2	3	4	5	6	7
10. There is a special person in my life who cares about my feelings.	1	2	3	4	5	6	7
11. My family is willing to help me make decisions.	1	2	3	4	5	6	7
12. I can talk about my problems with my friends.	1	2	3	4	5	6	7

Scale Reference:

Zimet GD, Dahlem NW, Zimet SG, Farley GK. The Multidimensional Scale of Perceived Social Support. *Journal of Personality Assessment* 1988;52:30-41.

Scoring Information:

To calculate mean scores:

Significant Other Subscale: Sum across items 1, 2, 5, & 10, then divide by 4.

Family Subscale: Sum across items 3, 4, 8, & 11, then divide by 4.

Friends Subscale: Sum across items 6, 7, 9, & 12, then divide by 4.

Total Scale: Sum across all 12 items, then divide by 12.

More information at:

http://gzimet.wix.com/mspss

Other MSPSS Scoring Options:

There are no established population norms on the MSPSS. Also, norms would likely vary on the basis of culture and nationality, as well as age and gender. I have typically looked at how social support differs between groups (e.g., married compared to unmarried individuals) or is associated with other measures (e.g., depression or anxiety). With these approaches you can use the mean scale scores.

If you want to divide your respondents into groups on the basis of MSPSS scores there are at least two ways you can approach this process:

1. You can divide your respondents into 3 equal groups on the basis of their scores (trichotomize) and designate the lowest group as low perceived support, the middle group as medium support, and the high group as high support. This approach ensures that you have about the same number of respondents in each group. But, if the distribution of scores is skewed, your low support group, for example, may include respondents who report moderate or even relatively high levels of support.

2. Alternatively, you can use the scale response descriptors as a guide. In this approach any mean scale score ranging from 1 to 2.9 could be considered low support; a score of 3 to 5 could be considered moderate support; a score from 5.1 to 7 could be considered high support. This approach would seem to have more validity, but if you have very few respondents in any of the groups, it could be problematic.

View publication stats
